# COVID-19 impacts on cross-border mobility of senior population between Shenzhen and Hong Kong

**DOI:** 10.3389/fpubh.2023.1285288

**Published:** 2023-11-20

**Authors:** Shi He, Caicheng Niu, Yue Wei, Yinger Cai, Wen Zhang, Yingbo Xiao, Jie Yin

**Affiliations:** ^1^School of Urban Planning and Design, Peking University Shenzhen Graduate School, Shenzhen, China; ^2^College of Urban and Environmental Sciences, Peking University, Beijing, China; ^3^Shenzhen Qianhai Construction and Investment Holding Group Co., Ltd., Shenzhen, China; ^4^Key Laboratory of Earth Surface System and Human-Earth Relations, Ministry of Natural Resources of China, Shenzhen, China

**Keywords:** COVID-19, mobility intervention policies, cross-border mobility (CBM), interrupted time series (ITS), senior population

## Abstract

The onset of the COVID-19 outbreak led to widespread adoption of mobility intervention policies, which were widely regarded as effective measures to control the spread of the virus. The initial pandemic wave, accompanied by the enforcement of mobility intervention policies, greatly changed human mobility patterns, especially cross-border mobility (CBM). This study investigates the impact of the first wave of the pandemic and related mobility intervention policies on the CBM of the senior population between Shenzhen and Hong Kong. Based on anonymous mobile phone trajectory data from 17 million devices active in Shenzhen spanning December 2019 to May 2020, we consider the implementation of mobility intervention policies during different stages of pandemic in both cities. We adopt interrupted time series (ITS) analysis to explore the causal effects of different mobility intervention policies on the CBM of older people between Hong Kong and Shenzhen. We find that most mobility intervention policies have a significant abrupt or gradual effect on the CBM of older people, especially in the 60–64 age group. As these policies neglect the mobility needs and characteristics among the senior groups, such as visiting relatives or friends and seeking medical treatment across borders, we suggest that more coordinated and integrated policies and measures are required to address the CBM needs of older people in Shenzhen and Hong Kong, especially in the post-pandemic era.

## 1 Introduction

Population aging means not only that the number of seniors is constantly increasing but also that current seniors are living longer and are more active than previous generations and may still be working or relaxing through travel after retirement, which involves crossing a wider geographical space and making more frequent long-distance trips than their predecessors (1). Existing research on the mobility and health of older people mainly focuses on their walking ability or their mobility using transportation tools within the city (2, 3). However, there is a need to bolster discussions regarding the cross-border mobility (CBM) of the older adults and its associated health ramifications, particularly during and after COVID-19 periods.

Cross-border travel demand among older people may reflect their physical health or psychological needs, with a correlation to their health outcomes. For example, some studies have found that daily CBM, as a form of relatively long-distance travel, can improve the physical and mental health of older people (4). Moreover, the older adults with a CBM demand are often a group of retired immigrants seeking better or more cost-effective medical services. For example, among a group of Japanese retired seniors who have lived in Thailand for a long time, 35% received health check-ups in Thailand within 12 months, 51.7% received check-ups in Japan, and 13.3% received check-ups in both countries, with frequent cross-border mobility often driven by healthcare needs due to health issues (5).

A limited number of studies have investigated factors affecting CBM in the senior population, including long-term and daily CBM. Regarding long-term CBM on the one hand, different regulations existing on either side of the border between two countries regarding retirement benefits, medical conditions, and long-term care have promoting, hindering, or strengthening effects on retirement migration mobility across the border (6). Based on textual data collected through in-depth interviews, Hsu et al. (4) found that the long-term CBM decisions of seniors between Hong Kong and mainland China are affected by both push-pull factors (e.g., living expenses and housing conditions) and life course determinants (e.g., past life experiences and previous housing history). Regarding short-term CBM on the other hand, some studies have found that culture and nature (e.g., visiting parks and rural or arts attractions), experience and adventure (e.g., traveling for enjoyment, escaping from one’s everyday routine, and novelty), and self-worth (e.g., visiting places where friends have not been or staying aligned with the travel experiences others engage in) are important correlates of choosing travel across borders (7).

Moreover, most CBM studies rely on qualitative research designs and methods with a relatively small sample size (8–10). Although qualitative analysis can reveal the underlying influencing factors of CBM, without quantitative analysis involving relatively large sample sizes, it often fails to test statistical relationships between the influencing factors and CBM, let alone the causality between them. Current studies on CBM based on quantitative analysis include descriptive statistics relating to mobility types or border crossers (11). These studies thus contribute to an evidence-based understanding of the occurrence of CBM and its health effects. Furthermore, against the background of the COVID-19 pandemic, the factors affecting the CBM of the older adults might have changed, influenced by mobility intervention policies during the pandemic, such as stay-at-home directives, lockdown measures, and travel bans. It is therefore also important to develop a causality-inference approach to track such time series treatment effects on the change in CBM.

To fill the abovementioned research gaps, this paper aims to examine how the first wave of the pandemic and related mobility intervention policies influenced the CBM of the senior population between Shenzhen and Hong Kong. Here, the border between Shenzhen and Hong Kong is a product of China’s “one country two systems” background. It is different from national boundaries as well as from boundaries between cities and regions.^1^ This study relies on anonymous mobile phone trajectory data from 17 million devices active in Shenzhen, providing a global perspective on seniors’ CBM. As abundant as the data is, it could be used to explain the causality between CBM and policies. We adopted an interrupted time series (ITS) research design to causally infer the effects of four stages of mobility intervention policies on the CBM flows of seniors between Hong Kong and Shenzhen.

This paper contributes twofold to the existing literature. First, it is among the first to estimate the causal effects of mobility intervention policies on seniors’ daily border crossings between Shenzhen and Hong Kong based on the ITS analysis. This helps to understand the impact of COVID-19 on older people’s CBM at different stages of the pandemic, when the two cities issued different mobility-related policies. This also helps us evaluate the effects of these mobility intervention policies between cities. Second, we focused on the mobility reactions among different senior population groups, ranging from early old age (55–59) and middle old age (60–64) to old age (above 65), considering that mobility intervention policies have varying impacts on CBM and related health outcomes among senior groups. This becomes especially pertinent given the likelihood of future pandemic-related mobility restrictions and the need to tailor policies to different senior population ‘unique cross border needs.

## 2 Literature review

### 2.1 Relationships between daily mobility and health of the senior population

Mobility is closely associated with the physical and mental health of older people. The relationship between mobility and the physical health of older people mainly includes two aspects: daily activities involving non-medical activities and medical visits. On the one hand, in non-medical activities, the frequency of using cars or cycling displayed a positive correction with physical activity scale, measured by the time allocated to leisure-time physical activity (12). Active travel in later life, such as walking or cycling, can reduce the risk of disease and mortality (13, 14). On the other hand, medical conditions, as a key component of the “legal gate,” play an important role in the CBM of older people. The legal gate refers to the daily rules and regulations at different levels, such as places and countries, that promote or hinder population mobility between one jurisdiction and another. It includes retirement benefits, medical conditions, and long-term care. The two aspects of the research constitute the main ways in which mobility affects the mental health of older people.

For older people in general, mobility has a positive impact on mental health, with reduced mobility potentially leading to increased feelings of loneliness and depression (15, 16). Research demonstrates that social participation is negatively associated with loneliness and life dissatisfaction; however, mobility inconvenience has been commonly mentioned as an obstacle to social participation (17, 18). Thus, mobility offers intrinsic benefits beyond meeting physical needs, notably in decreasing social isolation (19). This importance is amplified when considering a distinct demographic—seniors immigrants. Quantitative surveys in France and Switzerland have highlighted the adoption of a dual-residence strategy by many older immigrants, involving extended stays in their country of origin while designating their adult children’s residence as their primary one (20). This indicates that the essential role of CBM in meeting emotional needs and sustaining familial connections.

The decline in mobility during the COVID-19 lockdown has had a double impact on the physical and mental health of older people. According to Plagg et al. (21), the COVID-19 pandemic led to increased social isolation and loneliness among older people, which is associated with a higher incidence of vascular and nervous system diseases. Reduced opportunities for physical activity are also harmful to health. Therefore, both physical and mental health impacts can cause serious illness and premature death among older people. Under COVID-19 pandemic conditions, CBM was particularly restricted (22). A study by Huang et al. (23) revealed that Hong Kong seniors choose to live in Guangdong, Fujian, and other places due to factors such as social environment, consumption level, care services, and other conditions. However, they often need to return to Hong Kong for medical issues. Unfortunately, the COVID-19 lockdown resulted in drug shortages for Hong Kong seniors living outside the city. Despite efforts by the Hong Kong Government and the Hong Kong Federation of Trade Unions to assist in transporting drugs from Hong Kong to those seniors’ places of residence, on-site treatment was unavailable. Seniors patients with new symptoms or unstable conditions consequently still faced challenges in accessing adequate healthcare, which impacted their physical health.

### 2.2 Different mobility needs and health effects among different levels of senior groups

The senior population is commonly subdivided by age into early old age (65–75 years old), middle old age (75–85 years old), and old age (over 85 years old), although age is not the only division criterion (24). According to “The Third Report on Aging” by the German Federal Ministry (25), mobility plays an essential role in enhancing one’s quality of life in later life. It enables older individuals to maintain their activity level and lead fulfilling lives. However, as age-related health conditions may limit individual mobility, it is no longer taken for granted as it was in the past. Therefore, people attach growing importance to mobility as they progress in age (26). More specific results among the senior groups show that younger older people (65–74 years old) display robust mobility and have a travel frequency similar to that of 50–64-year-olds, while older people over 75 years old decline significantly. Concerning travel distance, as age increases, younger older people experience a significant decline compared with middle-aged people, while older people experience an even more pronounced decline (24).

A limited amount of literature exists on the differences in mobility needs and health effects among the senior groups. Relevant studies tend to focus on the individual mobility of older people in each age group, such as the ability to walk or climb stairs, or the impact of urban mobility on the health of older people. For instance, Musich et al. (2) found that the proportion of moderate and severe mobility limitation levels increased gradually with the age of older people. Specifically, at the moderate mobility limitation level, the proportions of 65–69-year-olds and 70–79-year-olds are similar, but at the severe level, the difference between the two groups increases significantly. Additionally, older people over 80 have significantly higher proportions of both types of mobility limitation levels than the other two groups. However, research focusing on more extensive mobility among the senior groups is currently lacking. From the perspective of urban mobility, Haustein and Siren (27) found that driving ability can meet the travel needs of older people and has a significant impact on their health. In Boschmann’s study (28) on the urban mobility of older people, it was found that individuals aged 65 to 74 had the highest independent driving ability and perceived mobility, while those aged 75 to 84 experienced a slight decline, and those over 85 declined significantly. However, the relationship between driving ability and mobility was not certain. Combining the findings from both studies reveals that the urban mobility of older individuals declines with age, and this decrease in mobility has a negative impact on their health.

During the COVID-19 pandemic, there was a significant decline in seniors’ mobility, which may have contributed to increased feelings of loneliness and social isolation, further affecting their health. Choe et al. (29) conducted a study in Hong Kong that further subdivided older individual differences (e.g., age and gender) and explored the influencing factors of daily mobility among the older individuals before and during the COVID-19 pandemic period. The study found that before COVID-19, the active travel behavior varied significantly among the senior groups. However, during the COVID-19 period, differences in older individuals’ characteristics (e.g., age and gender) did not significantly affect their active travel behavior. They attributed this phenomenon to the COVID-19 pandemic as an external condition that caused a decline in mobility. However, there remains a lack of in-depth exploration of this population’s CBM and the health effects among the senior groups during the COVID-19 pandemic.

In conclusion, although seniors have a strong demand for mobility, research on mobility and health among this group is currently limited to individual mobility or urban mobility. CBM also has an important impact on health; hence, further exploration is needed. Additionally, for special events, such as the COVID-19 pandemic, that restrict mobility, relevant literature does not subdivide policy changes according to each stage of older adults’ mobility and health. Research on the policy impacts at each stage will help formulate targeted policies among the senior groups, addressing their special mobility and health needs.

## 3 Research data and method

### 3.1 Research area and data source

This study focuses on the cross-border flow of seniors between Shenzhen and Hong Kong during the first wave of the COVID-19 pandemic (i.e., from December 2019 to May 2020). Shenzhen and Hong Kong are two adjacent cities located in the Guangdong-Hong Kong-Macao Greater Bay Area in southern China (30, 31). However, due to Shenzhen being part of mainland China and Hong Kong being a special administrative region (SAR) of China with an independent political and legal system, the flow of people between Shenzhen and Hong Kong is a typical cross-border flow. The two cities are connected by various means of transportation, including cross-border high-speed rail, subway, highway, and seaway. In addition, there are several border crossings between the two cities, including a total of 15 port crossings, such as Luohu Port, Futian Port, and Shenzhen Bay Port. Cross-border travelers are required to handle immigration and customs procedures at border checkpoints, and during the pandemic period, the freedom of population mobility at controlled ports decreased.

Shenzhen and Hong Kong have close relations, complement each other well, and have a large volume of cross-border flows. According to the Cross-boundary Travel Survey 2021 conducted by Hong Kong’s Planning Department, among travelers who traveled between Hong Kong and mainland China in 2021, those who usually reside in Hong Kong accounted for the largest proportion (63.3%) of the total number of travelers, while Shenzhen was the most popular destination for those residing in Hong Kong, accounting for 44.7% of total trips. People aged 25 to 54 years old conducted 68.2% of cross-border travel, while seniors aged 55 years old and above accounted for 21.1%. In terms of the purpose of travel from Hong Kong to mainland China, 55.4% of people visit relatives and friends, 21.9% are on business trips, and 6.4% travel for work purposes. In addition, other purposes, such as leisure and medical treatment, account for 16.3%. It is evident that the primary driver of cross-border flow is the need to visit relatives and friends.

In this study, we investigate the changes in the CBM of the senior population between Shenzhen and Hong Kong from December 1, 2019 to May 31, 2020. Our analysis is based on anonymous mobile phone trajectory data from 17 million devices active in Shenzhen, collected by China Unicom, one of China’s three major communication companies. This comprehensive dataset records a wide range of trips, tracking user locations during various activities, including phone calls, smartphone app use, internet use, and passive detection of movement activities, with updates every 30 min, enabling us to capture CBM data effectively. It includes details on the departure and arrival times of each recorded trip or movement, coupled with corresponding information about the origins and destinations of each anonymous user, as well as their demographic information, including age group and gender. Using the stay and move information, we identify cross-border travel patterns and define a CBM as a directed move from Shenzhen to Hong Kong or from Hong Kong to Shenzhen. By aggregating all the moves in one direction by volume, we can estimate the number of CBM flows.

### 3.2 Changing mobility intervention policies and trends of daily cross-border seniors during the COVID-19 pandemic

On January 19, 2020, China’s National Health Commission confirmed the first case of COVID-19 in Shenzhen. Shortly thereafter, on January 22, Hong Kong also reported its first case of COVID-19. From December 2019 to May 2020, there were 462 confirmed cases in Shenzhen and 1,084 in Hong Kong. Both cities implemented a series of mobility intervention policies to control the spread of the virus at different stages, including imposing travel bans, suspending the operation of port stations, enforcing compulsory quarantining, and providing information and guidance to the public, as listed in Table 1.

**Table 1:** The five stages with different virus-spread status and mobility intervention policies in Shenzhen and Hong Kong.

Stages	Time windows	Major mobility intervention policies and their mobility effects
Stage #0: Before COVID-19	2019/12/01–2020/01/14	Although Hong Kong initiated a severe response level to the COVID-19 pandemic on January 4, 2020, which included enhanced health measures for individuals arriving from Wuhan at the port, no policy was formulated at this stage to interfere with cross-border mobility between Shenzhen and Hong Kong.
Stage #1: Warning Stage	2020/01/15–2020/01/23	This stage commenced on January 15 when China’s National Health Commission issued the first edition of the COVID-19 diagnosis and treatment plan. Concurrently, the Hong Kong Food and Health Bureau declared that the virus’s limited human-to-human transmission could not be disregarded. Following the eruption of the pandemic in mainland China, on January 20 it was confirmed that the COVID-19 virus could be spread through human contact. This resulted in the implementation of more stringent policies in Shenzhen and Hong Kong to curb propagation of the virus, leading to a significant decrease in population mobility compared with that in the previous stage. In summary, the early measures for pandemic prevention and control centered on publicizing information and directing public opinion.
Stage #2: Freezing Stage	2020/01/24–2020/02/23	Since Guangdong’s first-level response to a major public health emergency on the evening of January 23, Shenzhen and Hong Kong raised pandemic prevention and control to the highest level, suspending the operation of 10 port control stations and keeping only three open. On February 8, Hong Kong mandated a 14-day quarantine for most mainland visitors. Through mandatory measures, Shenzhen and Hong Kong controlled population mobility, reduced contact, lowered the risk of close contact, and shortened the effective transmission period of the infected. As a result, there was minimal daily mobility between Shenzhen and Hong Kong. On February 9, Shenzhen required a health code for entry and exit, which further restricted senior from traveling due to digital pandemic prevention measures.
Stage #3: Adjustment Stage	2020/02/24–2020/04/29	Guangdong Province made the decision on February 24 to change from a first-level to a second-level response to a major public health emergency. Shenzhen’s pandemic prevention and control measures had been effective during this period, but Hong Kong had seen a surge in confirmed cases. As of midnight on March 19, all overseas visitors to Hong Kong, regardless of their residency status, were required to undergo 14 days of mandatory quarantine or medical surveillance.
Stage #4: Recovery Stage	2020/04/30–2020/05/31	Public services were restored by the Immigration Department of Hong Kong on April 30, and mandatory quarantine was lifted for cross-border students, businesspeople, and professionals. Shenzhen and Hong Kong efficiently managed the COVID-19 outbreak, and due to their close economic and social ties, they eased mandatory quarantine requirements for specific groups through a series of exemption policies. However, the travel aspirations of seniors were not fulfilled during this period.

**Figure 1:**
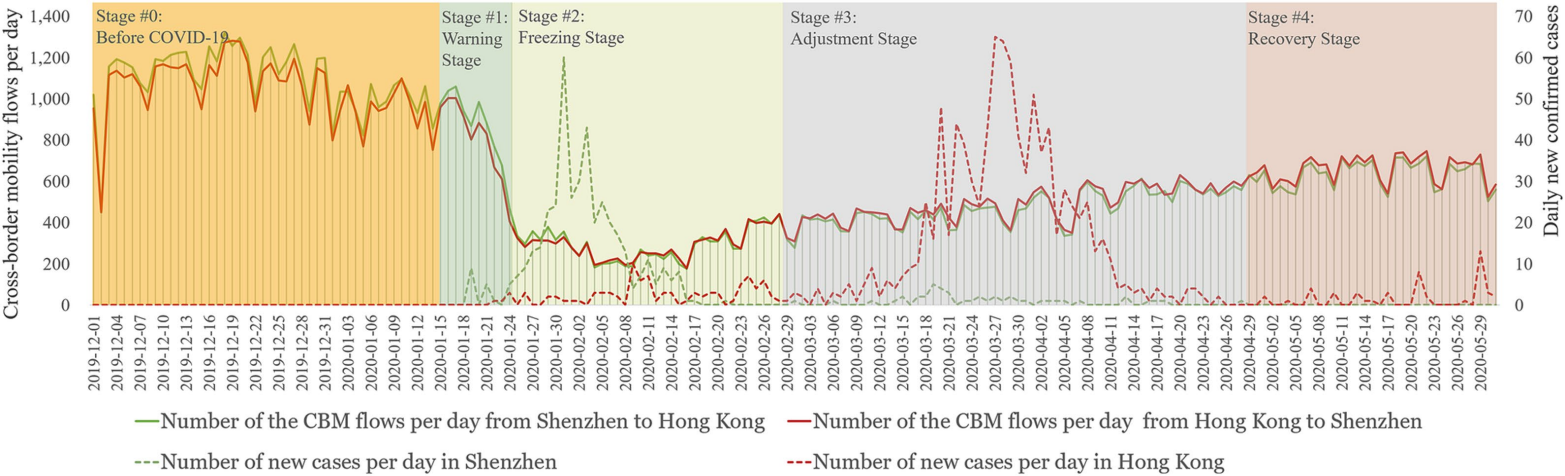
Seniors population flows between Hong Kong and Shenzhen during the first wave of the COVID-19 outbreak.

We divided the research period (from December 2019 to May 2020) into the following five stages based on the implementation of four stages of mobility intervention policies in both cities and the spread of the virus: the pre-COVID-19 stage (before January 15, 2020), the warning stage (January 15, 2020 to January 23, 2020), the freezing stage (January 24, 2020 to February 23, 2020), the adjustment stage (February 24, 2020 to April 29, 2020), and the recovery stage (April 30, 2020 to May 31, 2020). Figure 1 illustrates the changes in senior CBM flow volume from Shenzhen to Hong Kong and vice versa, as estimated based on the mobile phone trajectory big data. According to the figure, the changes in the CBM of seniors were consistent in both directions. From Hong Kong to Shenzhen, the average cross-border flow of seniors was 301 person-times/day before the pandemic. However, upon commencement of the warning stage, the average CBM flows of seniors declined to 222 person-times/day due to concerns about the human-to-human transmission of COVID-19. Subsequently, during the freezing stage, the CBM flow was constrained by Guangdong’s first-level response to a major public health emergency, resulting in a sharp drop to only 73 person-times/day. The freezing stage coincided with a surge in the number of cases of the virus in Shenzhen. On February 24, Guangdong Province decided to adjust the first-level response to a major public health emergency to a second-level response, and the adjustment stage saw a return of CBM flow to 139 person-times/day. During this stage, the number of cases in Shenzhen was under control, but the number of cases in Hong Kong rose. During the recovery stage, the number of cases in both Shenzhen and Hong Kong was under control, and Hong Kong enacted a favorable policy on CBM, leading to seniors crossing the border 192 person-times/day.

### 3.3 Research design and methods: an interrupted time series model

We adopted an interrupted time series (ITS) research design to causally infer the effects of four stages of mobility intervention policies on the CBM flows of seniors between Hong Kong and Shenzhen. An ITS is generally considered one of the most powerful quasi-experimental designs for causal inference because it uses long-term series observations of behavioral trends (32). Specifically, an ITS estimates policy intervention effects by predicting the trend of pre-intervention observations as counterfactuals and comparing the predicted trend with the actual trend of post-intervention observations (33, 34). ITS analyses have found broad applications across various fields, notably in public health, clinical research, and guideline implementation. Some studies also have used ITS designs to estimate the causal effects of mobility intervention policies on residents’ travel behavior and intra-urban mobility during the COVID-19 outbreak (35, 36). In this study, we employ ITS analysis to deduce both the abrupt and gradual effects of mobility interventions on CBM among the seniors. Additionally, for ITS analysis, we adopt the multilevel model. This model allows us to examine the seniors’ differences in the causal effects of mobility intervention policies.

Table 2 lists both dependent and independent variables for the ITS in accordance with the modelling specification provided below. For the dependent variable, we focused on the daily number of cross-border flows of seniors between Shenzhen and Hong Kong. We used mobility intervention policies for seniors at different stages as independent variables and gender, the Spring Festival holiday, COVID-19 cases in Hong Kong and Shenzhen, and the day of the week as control variables.

**Table 2:** Description of variables.

Variables		Description	Mean	St. dev.
Dependent variable				
*CBM*		CBM flow of seniors from Hong Kong to Shenzhen	30.15	21.05
Independent variables				
*Day*		Daily code for each day in the period of study	92	52.85
***Stage***	*Stage #1*	The intervention at the time point of the Warning Stage	0.05	0.22
	*Stage #2*	The intervention at the time point of the Freezing Stage	0.17	0.38
	*Stage #3*	The intervention at the time point of the Adjustment Stage	0.36	0.48
	*Stage #4*	The intervention at the time point of the recovery Stage	0.17	0.38
***Time***	*Time #1*	Daily code for each day after the intervention of the Warning Stage	0.20	1.04
	*Time #2*	Daily code for each day after the intervention of the Freezing Stage	2.54	6.73
	*Time #3*	Daily code for each day after the intervention of the Adjustment Stage	11.72	19.36
	*Time #4*	Daily code for each day after the intervention of the recovery Stage	2.71	7.04
***Age***	*Middle-old age dummy*	1: Seniors aged 60–64; 0: other old groups.	0.33	0.47
	*Old age dummy*	1: Seniors aged 65 and older; 0: other old groups.	0.33	0.47
***Control variables***				
*Case*	*HK_case*	New COVID-19 cases in Hong Kong	5.92	12.54
	*SZ_case*	New COVID-19 cases in Shenzhen	2.48	7.37
*SFHoliday*		Spring Festival holiday dummies (1: from January 24 to February 2, 2020; 0: other days.)	0.07	0.25
*Day of Week*		Weekly dummies, includes Monday to Saturday	0.14	0.35
Gender dummy		1: Female; 0: Male.	0.50	0.50

We adopted a multilevel mixed-effects model to estimate policy effects in an ITS design. We established a two-level model, with Level 1 comprising time-varying variables and Level 2 comprising socio-economic characteristics measured at the group level, such as age. The Level-1 model is as follows:

Level 1: Time-varying model


(1) $$\begin{array}{l} CBM_{it}=\beta_{oi}+\beta_{1i}Day_{it}+\beta_{2i}Stage_{t}+\beta_{3i}\left(Stage_{it}*Time_{it}\right)+\\ \beta_{4i}Case_{it}+\beta_{5i}SFHoliday_{it}+\\ \overset{6}{\underset{k=1}{\sum }}\beta_{\left(k+5\right)}{\left(Day\;ofWeek_{ij}\right)}_{k}+\varepsilon_{it}\;, \end{array}$$

where the subscript *i* represents the different population groups based on age and gender, subscript *t* represents time (varies from 1 to 183), and $CBM_{it}$ is the dependent variable in the ITS model, representing the CBM of each age group of seniors between Shenzhen and Hong Kong over the research period. Moreover, $Day_{it}$ is the daily code for each day in the period of study, equaling 1 for the first day and 183 for the last; $Stage_{it}$ represents the intervention of different time points, such as human-to-human transmission announcement, the highest-level response of the pandemic, which could be a vector of dummy variables; $Time_{it}$ is a vector of indicator variables that equals 0 before the interventions and counts from 1 (1,2, 3…) after the implementation of an intervention. Furthermore, we added several control variables, and $Case_{it}$ is a vector of new cases in Hong Kong (*HK_Case*) and Shenzhen (*SZ_Case*) at 183 days. We also added the Spring Festival holiday dummies (*SFHoliday*) and weekly dummies (*Day of Week*) to control for the possible holiday and weekly changes in CBM over the research period. Notably, due to COVID-19, the Spring Festival holiday in 2020 was extended from the regular seven days to 10 days (i.e., from January 24 to February 2, 2020). In the model, we centralized all control variables.

In detail, $\beta_{0}$ represents the CBM on the day before the research period (i.e., *day* 0), given that all other impact factors are controlled for, and $\beta_{1}$ is the average changing rate of CBM over time. Moreover, $\beta_{2}$ reflects the abrupt effects (change in level) of mobility intervention policies on the change in CBM, and $\beta_{3}$ is the change in trend from before COVID-19 to after interventions were introduced, thus referring to the causal effect in day-to-day trends or the change rate by day of each intervention effect. Finally, $\varepsilon_{it}$ is the error term.

According to the research question, we proposed that the causal effects of CBM intervention policies may differ among the senior groups. Therefore, the coefficient in Equation 1 is influenced by age, and its functional relationship is given by the Level-2 model as follows:

Level 2: Time-invariant model considering age difference among the senior group:


(2) $$\beta_{oi}=\varsigma_{00}+\varsigma_{01}Age_{i}+\varsigma_{02}Gender_{i}+\varsigma_{03}Age_{i}*Gender_{i}+\delta_{0i},$$

(3) $$\beta_{qi}=\varsigma_{q0}+\varsigma_{q1}Age_{i}+\delta_{qi},\left(q=1\sim 5\right)$$

The vector $Age_{i}$ consists of two dummy variables representing three different senior population groups: early old age (55–59), middle old age (60–64), and old age (65 and older), with early old age as the reference group. In Equation 2, $Gender_{i}$ is a dummy variable, where 0 represents male and 1 represents female; $\varsigma_{00}$ represents the mean of $\beta_{oi}$ parameters for males in the early-old-age group indicating the average number of CBM for males in this group; $\varsigma_{01}$ is the difference in the average CBM between males in the middle-old-age or old-age groups and males in the early-old-age group; $\varsigma_{02}$ is the difference in the means of CBM between males and females of early old age; $\varsigma_{03}$ reflects the gender difference in the means of CBM from early old age to middle old age or old age; $\varsigma_{q0}$ are the mean values of $\beta_{qi}$ in the different groups; and $\;\delta_{i}$ is a random term that represents unobserved idiosyncratic differences among groups.

## 4 Results

Table 3 presents the estimated results of the CBM of seniors from Hong Kong to Shenzhen in the ITS model. The results indicate significant differences in the CBM among the senior groups. Before the pandemic, CBM between Shenzhen and Hong Kong was dominant in the early-old-age group (90.66 person-times/day, *p* < 0.01), followed by the old-age group (50.88 person-times/day, *p* < 0.01) and the middle-old-age group (47.17 person-times/day, *p* < 0.01). At the same time, we observed a slight downward trend in the CBM of seniors: 0.11 person-times/day (*p* < 0.01).

**Table 3:** Estimated coefficients of variables in the ITS model (Dependent variable: daily CBM volume from Hong Kong to Shenzhen).

CBM		From Hong Kong to Shenzhen		
Variables		Coef.	St.Err.	*p* value
Pre-pandemic level and trend				
Day (the trend over time)		-0.11 ***	0.03	0.00
Early old age (55–59, as reference, the level on day 0)		90.66 ***	1.18	0.00
Middle old age (60–64, compared to early old age)		−43.49 ***	1.08	0.00
Old age (65+, compared to early old age)		−39.79 ***	1.08	0.00
**The abrupt effects on CBM flows during four stages among three senior groups**				
Stage #1: Warning Stage	Early old age (as reference, the level change in the Stage #1)	−5.60	3.42	0.10
	Middle old age (compared to early old age)	5.01	4.09	0.22
	Old age (compared to early old age)	5.40	4.09	0.19
Stage #2: Freezing Stage	Early old age (as reference, the level change in the Stage #2)	−54.68 ***	2.74	0.00
	Middle old age (compared to early old age)	24.58 ***	2.47	0.00
	Old age (compared to early old age)	21.87 ***	2.47	0.00
Stage #3: Adjustment Stage	Early old age (as reference, the level change in the Stage #3)	−44.55 ***	2.45	0.00
	Middle old age (compared to early old age)	16.95 ***	1.85	0.00
	Old age (compared to early old age)	19.15 ***	1.85	0.00
Stage #4: Recovery Stage	Early old age (as reference, the level change in the Stage #4)	−8.14 *	4.39	0.06
	Middle old age (compared to early old age)	4.83 **	2.44	0.05
	Old age (compared to early old age)	2.66	2.44	0.28
**The gradual effects on CBM flows during four stages among three senior groups**				
Stage #1 × Time #1: Warning Stage	Early old age (as reference, the slope change in the Stage #1)	−4.53 ***	0.68	0.00
	Middle old age (compared to early old age)	2.16 ***	0.84	0.01
	Old age (compared to early old age)	2.33 ***	0.84	0.01
Stage #2 × Time #2: Freezing Stage	Early old age (as reference, the slope change in the Stage #2)	0.04	0.13	0.78
	Middle old age (compared to early old age)	−0.09	0.13	0.49
	Old age (compared to early old age)	−0.03	0.13	0.81
Stage #3 × Time #3: Adjustment Stage	Early old age (as reference, the slope change in the Stage #3)	0.38 ***	0.05	0.00
	Middle old age (compared to early old age)	−0.01	0.04	0.88
	Old age (compared to early old age)	−0.13 ***	0.04	0.00
Stage #4 × Time #4: Recovery Stage	Early old age (as reference, the slope change in the Stage #4)	0.05	0.11	0.61
	Middle old age (compared to early old age)	0.05	0.12	0.71
	Old age (compared to early old age)	0.15	0.12	0.24
**Control Variables**				
Gender	Early old age (the difference between male and female)	−38.74 ***	0.96	0.00
	Middle old age (compared to early old age)	18.91 ***	0.96	0.00
	Old age (compared to early old age)	22.10 ***	0.96	0.00
Holiday		−0.57	1.07	0.60
HK_Cases		−0.03	0.02	0.16
SZ_Cases		−0.11 **	0.05	0.02
Monday		1.83 **	0.73	0.01
Tuesday		2.52 ***	0.73	0.00
Wednesday		3.28 ***	0.73	0.00
Thursday		2.90 ***	0.73	0.00
Friday		4.46 ***	0.73	0.00
Saturday		2.22 ***	0.73	0.00
Mean dependent var	30.152	SD dependent var.	21.053	
Number of obs	1,098	Chi-square	10502.763	
Prob > chi2	0.000	Akaike crit. (AIC)	7313.644	

Robust standard errors in parentheses: *** *p* < 0.01, ** *p* < 0.05, * *p* < 0.1.

**Figure 2:**
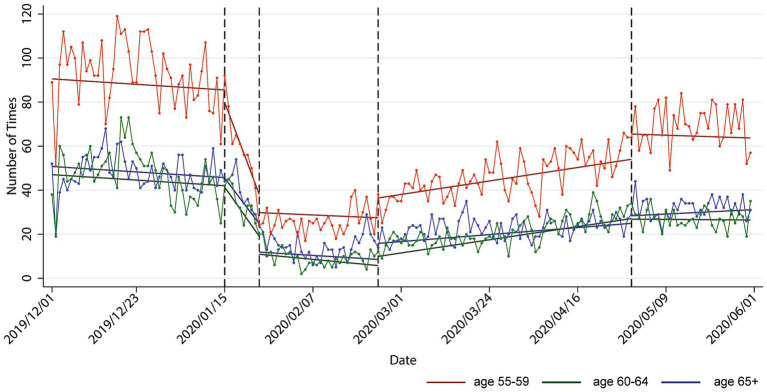
Estimated causal effects (abrupt and gradual) of the intervention policies on cross-border flows among the different senior groups.

Figure 2 illustrates the estimated effects of mobility intervention policies on the CBM of seniors from Hong Kong to Shenzhen during the research period in the ITS model. At the start of the warning stage on January 15, there was no significant instantaneous effect of early information dissemination and guidance about COVID-19 on CBM among seniors. However, the policies implemented during this stage, which encompassed information dissemination and the shaping of public opinion, led to a sustained decline effect, with a rate of-4.53 person-times/day among the early-old-age group (*p* < 0.01), −2.37 person-times/day among the middle-old-age group (*p* < 0.01), and-2.2 person-times/day among the old-age group (*p* < 0.01). Specifically, during the warning stage, the CBM of the early-old-age group decreased by 5.43% per day (calculated as [−4.53–0.11]/[90.66–0.11*46], where 46 represents the number of days counted from the initial date). For the middle-old-age group, CBM decreased by 5.91% per day, and for the old-age group, flows decreased by 5.05% per day. This indicates that the continuous impact of human-to-human transmission on reducing CBM is weakest for the old-age-group and strongest for the middle-old-age group.

During the freezing stage, which commenced on January 24, after Guangdong Province issued a first-level response, the CBM of seniors from Hong Kong to Shenzhen abruptly and significantly decreased. However, the immediate reduction effects of the first-level response on CBM varied among the senior groups. Particularly, the CBM of the middle-old-age group reduced immediately by 30.10 person-times (i.e., −54.68 + 24.58, *p* < 0.01). This reduction represents a substantial 73.20% decrease from the expected flow on January 24, based on the pre-pandemic trend (i.e., − 30.10/[90.66–43.49 − 0.11*55] = 73.20%, where 55 represents the number of days counted from the initial day). Similarly, the flow of the old-age group also drastically decreased by 73.20% (*p* < 0.01). By contrast, the flow of the early-old-age group experienced the smallest immediate reduction, at 64.61% (*p* < 0.01). These findings indicate that the first-level response policy had the strongest abrupt effect on CBM among the middle-old-age and old-age groups, while its effect on the early-old-age group was relatively weaker. Despite the tightening of intervention policies for CBM, some older population groups may still have had strong cross-border needs, such as medical treatment.

During the adjustment stage, which started on February 24, when Guangdong Province lowered its response level from the first to the second level, the CBM of the senior groups showed varying levels of immediate and gradual recovery. Figure 2 illustrates that there were significant abrupt reductions in the CBM of seniors from Hong Kong to Shenzhen on February 24 compared with the counterfactual scenario (i.e., if there were no pandemic). Specifically, the flow of the early-old-age group decreased instantaneously by 54.86% (calculated as 44.55/[90.66–0.11*86], where 86 represents the number of days counted from the initial date), that of the middle-old-age group decreased by 73.19% (i.e., [−44.55 + 16.95] = −27.60 person-times), and that of the old-age group decreased by 61.34% (i.e., [−44.55 + 19.15] = −25.40 person-times). However, during this stage, less reduction indicated a better recovery, which means that the early-old-age group exhibited the best recovery after the second-level response policy was issued, while the middle-old-age group experienced the lowest recovery. Furthermore, the negative effects of the second-level response policy were weakened over days with an upward change rate of 0.38 person-times in the early-old-age group (*p* < 0.01). The recovery in CBM among the old-age group was also reinforced by 0.25 person-times over days (i.e., 0.38–0.13, *p* < 0.01). Meanwhile, the trend of CBM recovery for the middle-old-age group compared with that of the early-old-age group did not show a significant difference.

During the recovery stage, which began after Hong Kong’s Immigration Department resumed public services on April 30, the CBM of seniors from Hong Kong to Shenzhen abruptly and significantly increased in the early-and middle-old-age groups compared with that in the previous adjustment stage. However, it has not yet returned to pre-pandemic levels. Compared with the counterfactual no-pandemic scenario, the CBM of the early-old-age group decreased by 11.01% (i.e., 8.14/[90.66–0.11*152, *p* < 0.01], where 152 represents the number of days counted from the initial day), and the CBM of the middle-old-age group decreased by 10.87% (*p* < 0.01). In addition, the decline effects for the old-age and early-old-age groups did not show a significant difference. According to the results, the middle-old-age group displayed the best recovery. Overall, these findings suggest that the older adults did not resume CBM promptly during the recovery stage, possibly due to slow information reception and limited familiarity with digital reporting appointments (37). Additionally, some older adults likely experienced anxiety or fear related to the pandemic, which could have also contributed to their hesitation to resume cross-border travel (38).

Table 4 lists the senior groups that were most and least affected by the changes in each period. Based on the results of the ITS model, the mobility intervention policies implemented during the warning and freezing stages demonstrated noteworthy negative impacts on CBM, whereas the policies enacted during the adjustment and recovery stages yielded positive effects. As a result, the assessment of policy impacts in the first two stages focuses on the extent of CBM decline among the senior groups, while the evaluation in the latter two stages measures the level of CBM recovery. For instance, the early-old-age group was less sensitive to the freezing stage policy but reacted more to the adjustment stage policy. The middle-old-age population, except for having the smallest response to the adjustment stage policy, was most affected by other policies. The old-age group was the most affected group during the freezing stage, but was the least affected by the warning and recovery stage policies. Moreover, we also found that while the CBM flows of female seniors were consistently smaller than those of men, these gender-based differences in CBM decreased with an increase in age. We also explored the sensitivity analysis to test the robustness of our results. The results of the ITS model from Shenzhen to Hong Kong are in the Supplementary Table S1.

**Table 4:** Comparison of policy impacts among the senior groups.

Time point of period change	Warning stage (2020/01/15–2020/01/23)	Freezing stage (2020/01/24–2020/02/23)	Adjustment stage (2020/02/24–2020/04/29)	Recovery stage (2020/04/30–2020/05/31)
Most Affected Age Group	Middle old age (age 60–64)	Middle old age (age 60–64), Old age (age 65+)	Early old age (age 55–59)	Middle old age (age 60–64)
Least Affected Age Group	Old age (age 65+)	Early old age (age 55–59)	Middle old age (age 60–64)	Old age (age 65+)

## 5 Conclusions and discussion

This study takes the CBM between Shenzhen and Hong Kong as an example and divides the research period from December 2019 to May 2020 into the following five stages based on the four implementation statuses of mobility intervention policies and the spread of the virus: the pre-COVID-19 stage, the warning stage, the freezing stage, the adjustment stage, and the recovery stage. Using anonymous mobile phone trajectory data from China Unicom at the Shenzhen-Hong Kong border, this study explores the causal effects of different mobility intervention policies among the senior groups and the disparities in these individuals’ responses to mobility intervention policies through ITS analysis.

Regarding CBM between Shenzhen and Hong Kong during the first wave of COVID-19 pandemic, there were age-related differences in how older individuals responded to mobility intervention policies in different stages. Overall, the 55–64 senior group was the most affected group by the impact of mobility intervention policies. This finding indicates that this group, compared with the 65+ senior group, had stronger adaptability to changes in mobility intervention policies, with a more substantial reduction in mobility during stringent restrictions and a subsequent increase in CBM following the implementation of recovery policies. This may be attributed to the fact that the age group of 55–64 relies more on CBM for various purposes, such as work, family visits, and leisure activities, which results in more pronounced responses to policy changes.

By contrast, the 65+ age group was less affected by mobility intervention policies. This tendency could be attributed to several factors. Firstly, it may be due to reduced sensitivity among individuals aged 65 and above in acquiring and comprehending new policies. Furthermore, their strong emphasis on physical health may lead them to prioritize conservative travel strategies aimed at reducing infection risks. In line with this, Choe et al. (29) similarly proposed that during the pandemic, more physically active older individuals were more likely to experience a reduction in their daily activity levels, while those of older age experienced a lesser decline in their mobility. Furthermore, Perracini et al. (38) also found that seniors under the age of 70 were significantly more affected by pandemic prevention and control policies compared to those aged 70 and older, attributing this difference to their higher employment rates.

This study provides the first estimation of the causal effects of mobility intervention policies on the daily CBM of older individuals between Shenzhen and Hong Kong. Although Shenzhen and Hong Kong established unified requirements and regulations in emergencies, effectively reducing CBM in different senior groups, there is still a lack of exploration into the differences in policy responses and activity patterns among the senior groups. Furthermore, this study underscores the importance of formulating age-specific policies, providing valuable insights for future research and policy development. For instance, since individuals aged 55–64 often have more frequent commutes and may frequently find themselves in caregiving and work roles, it is advisable for the governments of Shenzhen and Hong Kong to collaborate and ensure policy coordination to reduce inconveniences for older individuals commuting across the border. Additionally, heightened attention to psychological health, addressing feelings of solitude and social isolation due to mobility constraints, is essential. Furthermore, there is a need to provide tailored health services and information to individuals aged 65 and above. This will assist them in better comprehending and adapting to new policies and measures, as well as in accessing essential cross-border medical services and support.

However, the study has several limitations. Firstly, the data in this study originated from China Unicom. While it is one of the major telecom operators in mainland China and Hong Kong, it does not encompass data from other carriers. Hence, our analysis cannot represent all cross-border travel behaviors of the older adults in Shenzhen and Hong Kong. Although this study could determine how the CBM of the older adults is affected by mobility intervention policies, the underlying reasons and corresponding policies must still integrate multi-source data. Secondly, due to the data limitation, this study did not compare the CBM of the older adults with that of other age groups, which could reveal distinctive CBM traits among the older adults during the pandemic. Furthermore, although age is closely related to health issues and serves as a practical criterion for swift policy development in emergencies, it is essential to consider the underlying factors behind age, such as health conditions, basic living conditions, and medical records. Future studies should therefore aim to expand data sources, conduct cross-age comparisons, consider the factors associated with age, and explore the reasons and mechanisms behind the policies in depth. Such investigations would enhance current understanding of the factors affecting the CBM of the older adults and thus provide more targeted recommendations and guidance for future pandemic prevention and control as well as policymaking.

## Data availability statement

The data analyzed in this study is subject to the following licenses/restrictions: datasets generated and analyzed during the current study are not publicly available due to privacy restrictions but are available from the corresponding author on reasonable request. Requests to access these datasets should be directed to CN, niucc@stu.pku.edu.cn.

## Author contributions

SH: Conceptualization, Investigation, Writing – original draft. CN: Methodology, Supervision, Writing – original draft, Writing – review & editing. YW: Conceptualization, Data curation; Writing – original draft, Writing – review & editing. YC: Data curation; Software, Visualization, Writing – original draft, Writing – review & editing. WZ: Writing – original draft. YX: Writing – original draft. JY: Funding acquisition, Project administration, Supervision, Writing – original draft, Writing – review & editing.

## Footnotes

^1^Since Hong Kong’s return to the China in 1997, the demarcation between Hong Kong and mainland China has been officially referred to as a “boundary.” This term underscores the open border nature between Shenzhen and Hong Kong within a country, while highlighting the distinction between cities with different political systems within the same nation. However, in linguistic terms, “border” and “boundary” have only subtle semantic differences and are often used interchangeably. In this paper, we primarily employ “border” to denote the Shenzhen-Hong Kong boundary, consistent with common usage in related research.

## References

[1] Musselwhite C Holland C Walker I. The role of transport and mobility in the health of older people. J Transp Health. (2015) 2:1–4. doi: 10.1016/j.jth.2015.02.001

[2] Musich S Wang SS Ruiz J Hawkins K Wicker E. The impact of mobility limitations on health outcomes among older adults. Geriatr Nurs. (2018) 39:162–9. doi: 10.1016/j.gerinurse.2017.08.002, PMID: 28866316

[3] Thaithatkul P Chalermpong S Laosinwattana W Kato H. Mobility, activities, and happiness in old age: case of the elderly in Bangkok. Case Stud Transport Pol. (2022) 10:1462–71. doi: 10.1016/j.cstp.2022.05.010

[4] Hsu CHC Cai LA Wong KKF. A model of senior tourism motivations—anecdotes from Beijing and Shanghai. Tour Manag. (2007) 28:1262–73. doi: 10.1016/j.tourman.2006.09.015

[5] Miyashita Y Akaleephan C Asgari-Jirhandeh N Sungyuth C. Cross-border movement of older patients: a descriptive study on health service use of Japanese retirees in Thailand. Glob Health. (2017) 13:14. doi: 10.1186/s12992-017-0241-9, PMID: 28274263 PMC5343304

[6] Gehring A. Pensioners on the move: a ‘legal gate’ perspective on retirement migration to Spain. Population, space and place. Portico. (2015) 23:e2007. doi: 10.1002/psp.2007

[7] Patuelli R Nijkamp P. Travel motivations of seniors. Tour Econ. (2016) 22:847–62. doi: 10.1177/1354816616654257

[8] Karacan E. Coping with vulnerabilities in old age and retirement: cross-border mobility, family relations and social networks of German retirees in Alanya. Journal of Intergenerational Relationships. (2020) 18:339–57. doi: 10.1080/15350770.2020.1787048

[9] Legido-Quigley H Glinos IA Baeten R McKee M Busse R. Analysing arrangements for cross-border mobility of patients in the European Union: a proposal for a framework. Health Policy. (2012) 108:27–36. doi: 10.1016/j.healthpol.2012.07.001, PMID: 22871354

[10] Moret J. Typologising cross-border movements in post-migration life. European Somalis' Post-Migration Movements: Mobility Capital and Transnationalisation of Res. (2018) 51:2. doi: 10.1007/978-3-319-95660-2_2

[11] Järv O Aagesen HW Väisänen T Massinen S. Revealing mobilities of people to understand cross-border regions: insights from Luxembourg using social media data. Eur Plan Stud. (2022) 31:1754–75. doi: 10.1080/09654313.2022.2108312

[12] Tsunoda K Kitano N Kitagawa Y Tsuji T Soma Y Jindo T. Transportation mode usage and physical, mental and social functions in older Japanese adults. J Transp Health. (2015) 2:44–9. doi: 10.1016/j.jth.2014.10.003

[13] Hamer M Chida Y. Walking and primary prevention: a meta-analysis of prospective cohort studies. Br J Sports Med. (2008) 42:238–43. doi: 10.1136/bjsm.2007.039974, PMID: 18048441

[14] Sinnett D Williams K Chatterjee K Cavill N. Making the case for investment in the walking environment: A review of the evidence. Bristol: University of the West of England (2011).

[15] Edwards JD Perkins M Ross LA Reynolds SL. Driving Status and Three-Year Mortality Among Community-Dwelling Older Adults. J Gerontol Series A: Bio Sci Med Sci. (2009) 64A:300–5. doi: 10.1093/gerona/gln019 PMC2655033 19181711

[16] Ziegler F Schwanen T. ‘I like to go out to be energised by different people’: an exploratory analysis of mobility and wellbeing in later life. Ageing & Society. (2011) 31:758–81. doi: 10.1017/S0144686X10000498

[17] Currie G Delbosc A. Modelling the social and psychological impacts of transport disadvantage. Transportation. (2010) 37:953–66. doi: 10.1007/s11116-010-9280-2

[18] Goll JC Charlesworth G Scior K Stott J. Barriers to social participation among lonely older adults: the influence of social fears and identity. PLoS One. (2015) 10:e0116664. doi: 10.1371/journal.pone.0116664, PMID: 25706933 PMC4338142

[19] Mackett RL Thoreau R. Transport, social exclusion and health. Journal of Transport & Health. (2015) 2:610–7. doi: 10.1016/j.jth.2015.07.006

[20] Bolzman C Fibbi R Vial M. What to do after retirement? Elderly migrants and the question of return. J Ethn Migr Stud. (2006) 32:1359–75. doi: 10.1080/13691830600928748

[21] Plagg B Engl A Piccoliori G Eisendle K. Prolonged social isolation of the elderly during COVID-19: between benefit and damage. Arch Gerontol Geriatr. (2020) 89:104086. doi: 10.1016/j.archger.2020.104, PMID: 32388336 PMC7196375

[22] Zhang W Gong Z Niu C Zhao P Ma Q Zhao P. Structural changes in intercity mobility networks of China during the COVID-19 outbreak: A weighted stochastic block modeling analysis. Com Environ Urban Sys. (2022) 96:101846. 10.1016/j.compenvurbsys.2022.101846 PMC9194079 35719244

[23] Huang G Ma Y Peng Z. Cross-border medical services for Hong Kong's older adults in mainland China: the implications of COVID-19 for the future of telemedicine In: Miller EA, editor. The COVID-19 pandemic and older adults: Routledge (2022). 217–29. 10.1080/08959420.2021.1925051 34039233

[24] Klein-Hitpaß A Lenz B. Mobility of the elderly–facts and projections. Demography and Infrastructure: National and Regional Aspects of Demographic Change. (2011) 51:9. doi: 10.1007/978-94-007-0458-9_9

[25] BMFSFJ. Dritter Altenbericht der Bundesregierung. Berlin: German Federal Ministry (2001).

[26] Blume V Follmer R Kalinowska D Kloas J. Demographischer Wandel und räumliche Mobilität: Einstellungen der Bevölkerung. Urteile von Experten DIW Wochenbericht. (2005) 72:769–75.

[27] Haustein S Siren AK. Seniors’ unmet mobility needs – how important is a driving licence? J Transp Geogr. (2014) 41:45–52. doi: 10.1016/j.jtrangeo.2014.08.001

[28] Boschmann E. Daily urban trip mobility and perceptions of mobility among older adults in the Denver (USA) region. J Aging Soc Change. (2020) 10:33–52. doi: 10.18848/2576-5310/cgp/v10i02/33-52

[29] Choe EY Du Y Sun G. Decline in older adults’ daily mobility during the COVID-19 pandemic: the role of individual and built environment factors. BMC Public Health. (2022) 22:2317. doi: 10.1186/s12889-022-14780-8, PMID: 36503494 PMC9742036

[30] Zhang W Fang C Zhou L Zhu J. Measuring megaregional structure in the Pearl River Delta by mobile phone signaling data: A complex network approach. Cities. (2020) 104:102809.

[31] Zhang W Zhao P Niu C Thill JC Zhu J. City networks and clusters as expressed in Chinese and Japanese languages: A multiscale network analysis with language-sensitive webpage big data. Cities. (2023) 141:104502.

[32] Reichardt CS. Quasi-experimentation: A guide to design and analysis. New York: Guilford Publications (2019).

[33] Liu C Zhang W. Social and spatial heterogeneities in COVID-19 impacts on individual’s metro use: A big-data driven causality inference. Appl Geography. (2023) 155:102947. 10.1016/j.apgeog.2023.102947 PMC10070784 37035417

[34] Zhang W Li J. A quasi-experimental analysis on the causal effects of COVID-19 on urban park visits: The role of park features and the surrounding built environment. Urban Forestry Urban Greening. (2023) 82:127898. 36915824 10.1016/j.ufug.2023.127898 PMC9988312

[35] Niu C Zhang W. Causal effects of mobility intervention policies on intracity flows during the COVID-19 pandemic: the moderating role of zonal locations in the transportation networks. Comput Environ Urban Syst. (2023) 102:101957. doi: 10.1016/j.compenvurbsys.2023.101957, PMID: 36938101 PMC10011038

[36] Zhang W Ning K. Spatiotemporal heterogeneities in the causal effects of mobility intervention policies during the COVID-19 outbreak: a spatially interrupted time-series (SITS) analysis. Ann Am Assoc Geogr. (2023) 113:1112–34. doi: 10.1080/24694452.2022.2161986

[37] Yang C Ma CY Wang J Zhou Y. Cross-border ageing in China’s Greater Bay Area in the digital age: a comparative study of mobile application adoption by Hong Kong older migrants and local older adults in Shenzhen. Trans Planning and Urban Res. (2023) 2:149–66. doi: 10.1177/27541223221150653

[38] Perracini MR De Amorim JSC Lima CA Da Silva A Trombini-Souza F Pereira DS. Impact of COVID-19 pandemic on life-space mobility of older adults living in Brazil: REMOBILIZE study. Front Public Health. (2021) 9:313. doi: 10.3389/fpubh.2021.643640, PMID: 33898378 PMC8062747

