# Supplementary material for: COVID-19 impacts on cross-border mobility of senior population between Shenzhen and Hong Kong
Source: Front Public Health. 2023 Nov 20;11:1285288. doi: 10.3389/fpubh.2023.1285288 (PMC10694502; doi:10.3389/fpubh.2023.1285288)
Supplement: Supplementary file 1 [file Table_1.DOCX]

Supplementary Material

The results from the two models in Table 3 and Supplementary Table 1 were largely consistent, demonstrating the robustness of the results. Thus, this study primarily focuses on CBM from Hong Kong to Shenzhen. A slight inconsistency between the models occurred in the warning stage – mobility intervention policies had similar effects on CBM in both directions, but were statistically significant only for Shenzhen to Hong Kong at *p* < 0.05.

**Supplementary Table 1.** Estimated results of core variables in the ITS model (dependent variable: CBM from Shenzhen to Hong Kong).

| **CBM** | | | | **From Shenzhen to Hong Kong** | | |
| --- | --- | --- | --- | --- | --- | --- |
| **Variables** | | | | **Coef.** | **St.Err.** | ***p* value** |
| **Pre-pandemic level and trend** | | | | | | |
| Day (the trend over time) | | | | -0.12 *** | 0.03 | 0.00 |
| Early old age (55-59, as reference, the level on day 0) | | | | 93.30 *** | 1.24 | 0.00 |
| Middle old age (60-64, compared to early old age) | | | | -44.51 *** | 1.14 | 0.00 |
| Old age (65+, compared to early old age) | | | | -40.25 *** | 1.14 | 0.00 |
| **The abrupt effects on CBM flows during four stages among three senior groups** | | | | | | |
| Stage #1:  Warning Stage | Early old age (as reference, the level change in the Stage #1) | | | -8.60 ** | 3.60 | 0.02 |
|  | Middle old age (compared to early old age) | | | 3.38 | 4.32 | 0.43 |
|  | Old age (compared to early old age) | | | 3.94 | 4.32 | 0.36 |
| Stage #2:  Freezing Stage | Early old age (as reference, the level change in the Stage #2) | | | -55.98 *** | 2.88 | 0.00 |
|  | Middle old age (compared to early old age) | | | 24.69 *** | 2.60 | 0.00 |
|  | Old age (compared to early old age) | | | 22.15 *** | 2.60 | 0.00 |
| Stage #3: Adjustment Stage | Early old age (as reference, the level change in the Stage #3) | | | -47.16 *** | 2.59 | 0.00 |
|  | Middle old age (compared to early old age) | | | 18.66 *** | 1.95 | 0.00 |
|  | Old age (compared to early old age) | | | 20.28 *** | 1.95 | 0.00 |
| Stage #4: Recovering Stage | Early old age (as reference, the level change in the Stage #4) | | | -11.90 ** | 4.63 | 0.01 |
|  | Middle old age (compared to early old age) | | | 7.39 *** | 2.57 | 0.00 |
|  | Old age (compared to early old age) | | | 5.66 ** | 2.57 | 0.03 |
| **The gradual effects on CBM flows during four stages among three senior groups** | | | | | | |
| Stage #1×Time #1:  Warning Stage | Early old age (as reference, the slope change in the Stage #1) | | | -3.75 *** | 0.72 | 0.00 |
|  | Middle old age (compared to early old age) | | | 2.32 *** | 0.88 | 0.01 |
|  | Old age (compared to early old age) | | | 2.58 *** | 0.88 | 0.00 |
| Stage #2×Time #2:  Freezing Stage | Early old age (as reference, the slope change in the Stage #2) | | | -0.01 | 0.13 | 0.94 |
|  | Middle old age (compared to early old age) | | | -0.02 | 0.14 | 0.89 |
|  | Old age (compared to early old age) | | | -0.01 | 0.14 | 0.92 |
| Stage #3×Time #3:  Adjustment Stage | Early old age (as reference, the slope change in the Stage #3) | | | 0.38 *** | 0.05 | 0.00 |
|  | Middle old age (compared to early old age) | | | 0.00 | 0.04 | 0.98 |
|  | Old age (compared to early old age) | | | -0.13 *** | 0.04 | 0.00 |
| Stage #4×Time #4:  Recovering Stage | Early old age (as reference, the slope change in the Stage #4) | | | 0.13 | 0.11 | 0.26 |
|  | Middle old age (compared to early old age) | | | 0.02 | 0.13 | 0.90 |
|  | Old age (compared to early old age) | | | 0.08 | 0.13 | 0.57 |
| **Control Variables** | | | |  | | |
| **Gender** | Early old age (the difference between male and female) | | | -39.50 *** | 1.01 | 0.00 |
|  | Middle old age (compared to early old age) | | | 18.39 *** | 1.01 | 0.00 |
|  | Old age (compared to early old age) | | | 22.13 *** | 1.01 | 0.00 |
| Holiday | | | | -0.69 | 1.13 | 0.54 |
| HK_Cases | | | | -0.03 | 0.02 | 0.11 |
| SZ_Cases | | | | -0.15 *** | 0.05 | 0.00 |
| Monday | | | | 1.86 ** | 0.77 | 0.02 |
| Tuesday | | | | 2.81 *** | 0.77 | 0.00 |
| Wednesday | | | | 3.87 *** | 0.77 | 0.00 |
| Thursday | | | | 2.77 *** | 0.77 | 0.00 |
| Friday | | | | 4.58 *** | 0.77 | 0.00 |
| Saturday | | | | 2.02 *** | 0.77 | 0.01 |
| Mean dependent var | | 30.461 | SD dependent var | | | 21.536 |
| Number of obs | | 1098 | Chi-square | | | 9829.828 |
| Prob > chi2 | | 0.000 | Akaike crit. (AIC) | | | 7429.130 |

Robust standard errors in parentheses: *** *p* < 0.01, ** *p* < 0.05, * *p* < 0.1.
